# Optimizing *Chlorella vulgaris* Cultivation to Enhance Biomass and Lutein Production

**DOI:** 10.3390/foods13162514

**Published:** 2024-08-12

**Authors:** Kangping Wu, Jiangling Lai, Qi Zhang, Yunpu Wang, Xian Cui, Yuhuan Liu, Xiaodan Wu, Zhigang Yu, Roger Ruan

**Affiliations:** 1 State Key Laboratory of Food Science and Resources, Engineering Research Center for Biomass Conversion, Ministry of Education, Nanchang University, Nanchang 330047, China; wukangping@gnust.edu.cn (K.W.); 417900210044@email.ncu.edu.cn (J.L.); wangyunpu@ncu.edu.cn (Y.W.); cuixian@ncu.edu.cn (X.C.); wuxiaodan@ncu.edu.cn (X.W.); 2 School of Resources and Civil Engineering, Gannan University of Science and Technology, Ganzhou 341000, China; 3 College of Food Science and Technology, Nanchang University, Nanchang 330047, China; 4 Australian Centre for Water and Environmental Biotechnology (Formerly AWMC), The University of Queensland, Brisbane, QLD 4072, Australia; zhigang.yu@uq.net.au; 5 Center for Biorefining and Department of Bioproducts and Biosystems Engineering, University of Minnesota, St. Paul, MN 55108, USA; ruanx001@umn.edu

**Keywords:** *Chlorella vulgaris*, culture conditions, biomass production, lutein production

## Abstract

Lutein is widely used in medicine, health care, and food processing due to its antioxidant effects; however, it is difficult for the traditional extraction of lutein using marigolds to meet the increasing market demand for lutein. To achieve high-efficiency lutein production, we investigated the effects of different conditions on the biomass accumulation and lutein yield of *Chlorella vulgaris*. The optimized cultivation conditions include mixotrophic cultivation using sodium nitrate as a nitrogen source, maintaining a total-organic-carbon-to-total-nitrogen ratio of 12:1, a total-nitrogen-to-total-phosphorus ratio of 10:1, and lighting duration of 24 h. The results of the study indicated that under these specific conditions, *Chlorella vulgaris* attained a final biomass concentration, biomass productivity, and growth yield of 6.08 g·L^−1^, 1.00 g·L^−1^·d^−1^, and 1.67 g biomass/g TOC, respectively. Additionally, the concentrations of total chlorophyll, carotenoid, lutein, and protein reached 139.20 mg·L^−1^, 31.87 mg·L^−1^, 15.02 mg·L^−1^, and 2.17 g·L^−1^, respectively, and the content of lutein reached 2.47 mg·g^−1^. This study supplies a theoretical basis for the industrial application of lutein production using *Chlorella vulgaris*.

## 1. Introduction

Lutein is a xanthophyll carotenoid present in higher plants and other photoautotrophic organisms such as microalgae [1]. It has been proven to play a notable part in the amelioration and treatment of ocular diseases such as cataracts and age-related macular degeneration [2]. Lutein intake also has a better inhibitory effect on the development of cancer [3]. Due to its potent antioxidant properties [4], lutein is widely available as feed additives and functional supplements to mitigate photooxidative damage, with significant applications in the fields of healthcare and food processing [5].

The human body is incapable of synthesizing lutein via chemical processes [6]. Petals from marigold flowers serve as a primary ingredient in the manufacturing process of commercially accessible lutein supplements [7]. Nevertheless, these petals contain a significantly low lutein content, and their growth is seasonally limited. Marigold flowers necessitate arable land usage and demand skilled labor for processing and extraction [8], leading to elevated production costs. Consequently, there is an urgent need to discover a cost-effective and economically viable substitute for lutein in commercial production.

In comparison to marigolds, the utilization of microalgae as the lutein feedstock presents numerous advantages. Firstly, microalgae can be used to manufacture a wide range of valuable compounds, such as lipids, lutein, and protein. Secondly, microalgae cultivation is not restricted by seasonal changes and allows for cultivation on saltwater and non-cultivated land [7]. Additionally, microalgae have a short growth cycle, enabling continuous harvesting throughout the year [1]. Furthermore, compared to marigold petals, microalgae have a higher lutein content. The lutein content of marigold petals generally ranges from 0.22 to 0.98 g·kg^−1^ and is within 0.10 wt% by weight of the flowers [7,9], whereas the lutein content (converted to g/kg biomass) of microalgae is in the range of 3.40–7.60 g·kg^−1^, which equals 0.34–0.76 wt% [10]. Therefore, microalgae are being considered as a prospective replacement source for lutein production.

To enhance the commercial viability of microalgae-based lutein production, it is imperative to achieve a substantial increase in lutein productivity. To obtain an increased lutein productive rate, lutein content and biomass productivity should be increased by optimizing the culture conditions. According to recent relevant research [4,11], a variety of cultivation factors were considered on a global scale, including nutrient patterns, carbon sources, light intensities, and light/dark cycles [12]. Chen, et al. [13] investigated the strategy of lutein production in a heterotrophic culture of *Chlorella*, exploring various carbon–nitrogen ratios and establishing that a ratio of 10:1 led to the highest lutein productivity of 4.88 mg·L^−1^·d^−1^. Shi, et al. [14] demonstrated that the growth conditions, such as the culture mode, carbon source, and nitrogen availability, exerted a substantial influence on both lutein accumulation and microalgae biomass productivity.

Therefore, in this study, the aim is to optimize culture conditions for high lutein (main product) and protein (byproduct) production in *Chlorella vulgaris*. The effects of various culture modes, the nitrogen source, the total-organic-carbon (TOC)-to-total-nitrogen (TN) ratio (TOC/TN), the total-nitrogen (TN)-to-total-phosphorus (TP) ratio (TN/TP), and the lighting duration on the growth of *C. vulgaris* and the accumulation of total chlorophyll, carotenoids, lutein, and protein were explored via an orthogonal experiment and a single factor experiment. The results obtained in this study present an optimal approach for accumulating biomass and producing lutein in *C. vulgaris*. These findings provide valuable insights for evaluating the use of microalgae as a feedstock for the full-scale production of lutein. This has significant theoretical and practical implications for advancing the industrial applications of *C. vulgaris* in lutein production.

## 2. Materials and Methods

### 2.1. Microalgae Strain and Seed Culture

The microalgae used in this study was *Chlorella vulgaris* FACHB-31, purchased from the Institute of Hydrobiology, Chinese Academy of Sciences, PR China.

Before formal inoculation, *C. vulgaris* was initially cultured in BG-11 medium at 30 °C under continuous illumination of 10,000 lux for 3–4 days. Subsequently, the culture was moved to a centrifuge tube that had been pre-sterilized and weighed. It was then subjected to centrifugation at 8000 rpm and 4 °C for a duration of 5 min. The resulting precipitate was washed twice with sterile deionized water and finally used as the inoculum for the subsequent experiment. The composition of BG-11 is listed in Table S1.

### 2.2. Experimental Design

The basal culture medium for heterotrophic and mixotrophic cultivation of *C. vulgaris* were both BG-11-supplemented with glucose, as indicated in Table S1. Subsequent optimization experiments were conducted by adjusting the composition of the basal medium. The pH of all media was adjusted to 7.00. Then, 300 mL of each medium was transferred into 500 mL flasks and sterilized at 121 °C for 20 min. The inoculation concentration for each group remained consistent at 2.5 ± 0.5 × 10^6^ cells·mL^−1^. *C. vulgaris* inoculation procedure is detailed in Section 2.1. Each group’s cultures were placed in a shaking incubator (120 rpm) and maintained at 30 °C, with the heterotrophic group maintained in darkness and the mixotrophic group exposed to a continuous illumination of 10,000 lux for 6 days. In order to investigate the influence of culture modes and various nitrogen sources on lutein production performance, the culture was grown in six groups: heterotrophic + sodium nitrate (HSN), heterotrophic + ammonium acetate (HAA), heterotrophic + urea (HU), mixotrophic + sodium nitrate (MSN), mixotrophic + ammonium acetate (MAA), and mixotrophic + urea (MU). The consistency of the TN concentration was controlled while using sodium nitrate, ammonium acetate, and urea as nitrogen sources, with heterotrophic and mixotrophic cultivation, respectively. The effect of the initial TOC/TN ratio (3:1, 6:1, 9:1, 12:1, and 18:1) of the media on *C. vulgaris* cultivation and lutein accumulation was investigated by adjusting the additional amount of nitrogen. And the effect of the initial TN/TP ratio (5:1, 10:1, 25:1, 50:1, and 80:1) of the media was studied by adjusting the additional amount of phosphorus (K_2_HPO_4_). Additionally, to evaluate the influence of the lighting duration, four different lighting durations (6, 12, 18, and 24 h) were applied during cultivation. All experimental conditions (including TOC and TP concentrations, etc.) remained consistent throughout the study except for the specific treatment being assessed in order to determine its individual effect on *C. vulgaris*. A total of 10 mL of samples from each culture flask were collected every 24 h for subsequent analysis during the entire cultivation process. After the cultivation, cultures were subjected to centrifugation at a speed of 8000 rpm for a duration of 10 min. The resulting precipitates were then washed twice with deionized water before being placed in petri dishes and freeze-dried at −80 °C for further analysis. The specific compositions of the adjusted medium in different treatments are shown in Tables S2–S4.

### 2.3. Analytical Methods

#### 2.3.1. Biomass Analysis

The concentration of microalgal biomass was ascertained through the measurement of the absorbance of the microalgal suspension at 680 nm (OD680) [15].

The biomass concentration of *C. vulgaris* was linearly correlated with OD680, and the standard calibration curve is plotted in Figure S1. The biomass concentration (g·L^−1^) was calculated according to Equation (1).

(1) $$Biomass\;concentration\left(g\cdot L^{-1}\right)=0.2549\times OD_{680}-0.0089\;(R^{2}=0.9973)$$

The growth kinetic parameters of *C. vulgaris* were calculated using the methodology established by Cai, et al. [16] as follows:

The maximum specific growth rate (*μ*_max_, day^−1^) of *C. vulgaris* was calculated according to Equation (2).

(2) $$\mu_{\max}\left({\mathrm{day}}^{-1}\right)=\frac{\ln W_{2}-\ln W_{1}}{t}$$

where *W*_1_ and *W*_2_ denote the biomass concentration (g·L^−1^) at the onset and conclusion of the exponential growth phase, correspondingly, while *t* represents the duration of this phase in days.

The generation time (*T*, days) was calculated according to Equation (3).

(3) $$T\left(\mathrm{days}\right)=\frac{\ln2}{\mu_{\max}}$$

The biomass productivity (*P*, g·L^−1^·day^−1^) was calculated according to Equation (4).

(4) $$P\left(g\cdot L^{-1}\cdot {\mathrm{day}}^{-1}\right)=\frac{W_{6}-W_{0}}{6}$$

where *W*_0_ and *W*_6_ denote the biomass concentration (g·L^−1^) at days 0 and 6, respectively.

The growth yield (*G*, g biomass/g TOC) was calculated according to Equation (5).

(5) $$G\left(g_{\mathrm{Biomass}}{/g}_{\mathrm{TOC}}\right)=\frac{W_{6}-W_{0}}{TOC_{6}-TOC_{0}}$$

where *TOC*_0_ and *TOC*_6_ denote the total organic carbon (TOC) concentrations (g·L^−1^) at days 0 and 6, respectively.

#### 2.3.2. Nutrient Consumption Analysis

The concentration of reducing sugar in the solution was measured using the 3,5-dinitrosalicylic acid (DNS) method in an alkaline environment, in accordance with the methodology described by Wang, et al. [17].

The algal samples of 3 mL were collected daily from each flask and centrifuged at a speed of 10,000 rpm for 4 min. The resulting supernatant underwent filtration using a membrane with a pore size of 0.45 μm. Subsequently, appropriate dilution was performed on the filtrate before determining the concentrations of total inorganic carbon (IC), TOC, and TN in the filtrate were determined using a TOC/TN analyzer (Multi N/C 3100, Analytik Jena AG, Jena, Germany).

#### 2.3.3. Value-Added Compounds Production Analysis

The total chlorophyll and carotenoid content of the samples were characterized by a methanol extraction method using a spectrophotometer (UV-9000, Metash, Shanghai, China) with reference to Zhou, et al. [18]. The remaining algal slime in Section 2.3.2 was washed and mixed with 99.80% (*v*/*v*) methanol and kept at 4 °C in dark conditions for 24 h until the slime turned white. The absorbances at 665.2 (*A*_665.2_), 652.4 (*A*_652.4_), and 470 (*A*_470_) nm were determined after centrifugation. The concentrations of chlorophyll *a* (*Chl-a*), chlorophyll *b* (*Chl-b*), and carotenoids were calculated according to Equations (6)–(8), respectively.

(6) $$Chl\text{-}a\left(\mathrm{mg}\cdot L^{-1}\right)=16.72A_{665}-9.16\times A_{652.4}\;$$

(7) $$Chl\text{-}b\left(\mathrm{mg}\cdot L^{-1}\right)=34.09A_{652.4}-15.28A_{665.2}\;$$

(8) $$Carotenoid\left(\mathrm{mg}\cdot L^{-1}\right)=\left(1000A_{470}-1.63Chl\text{-}a-104.9Chl\text{-}b\right)/221$$

Lutein content was determined using HPLC (1260, Agilent Technologies, Lake Forest, CA, USA) with the external standard method as described by Wu, et al. [19]. Lutein was extracted from freeze-dried algal powder using dimethyl sulfoxide (DMSO) as the extracting solvent. The HPLC conditions employed were as follows: a separation column of Eclipse Plus C18 (250 mm × 4.6 mm, 5 μm) was employed, and the mobile phase consisted of a mixture containing tetrahydrofuran, methanol, and acetonitrile in a volumetric ratio of 5:45:50. The UV detector was used for detection at a wavelength of 446 nm. The column temperature was set to 30 °C, and the flow rate was maintained at 1.0 mL·min^−1^. The protein content in algal powder was measured by the Kjeldahl method following the method described by [20].

### 2.4. Statistical Analysis

All experiments were performed in triplicate, and the data were presented as the mean ± standard deviation. One-way analysis of variance (ANOVA) and Duncan’s test was carried out using IBM SPSS Statistics 20 (SPSS Inc., Chicago, IL, USA), with statistical significance defined as *p* < 0.05.

## 3. Results and Discussion

### 3.1. Effect of Culture Mode and Nitrogen Source

**Figure 1:**
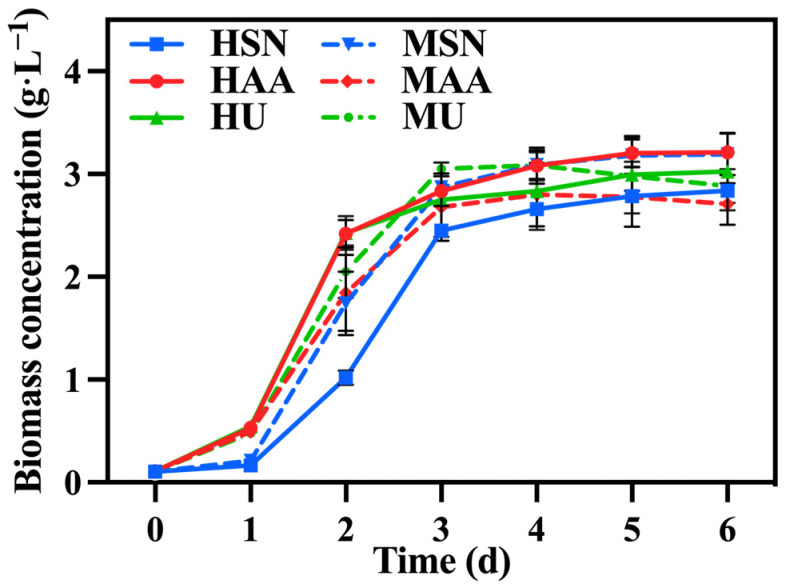
The biomass concentration in different groups with varying nitrogen sources and culture modes.

Figure 1 illustrates the change in biomass concentration under different treatments. Initially, *C. vulgaris* in the groups supplemented with ammonium acetate (HAA, MAA) exhibited a significantly higher growth rate relative to the group supplemented with sodium nitrate (HSN, MSN) since it can directly assimilate ammonium nitrogen as the favored nitrogen [21]. And nitrate nitrogen can only be utilized after its conversion into ammonium nitrogen, which is catalyzed by reductase [22]. The biomass of *C. vulgaris* in the group supplemented with urea (HU, MU) was comparable to that of the group supplemented with ammonium acetate (HAA, MAA) on the first day, probably due to the decomposition of urea into ammonium nitrogen during sterilization. However, this advantage diminished over time. This trend can be attributed to the substantial production of reductase from the groups supplemented with sodium nitrate (HSN, MSN), enabling *C. vulgaris* to also utilize nitrate nitrogen at an accelerated rate. Table 1 also shows that there was no significant difference in biomass concentration and biomass productivity among the various groups.

**Figure 2:**
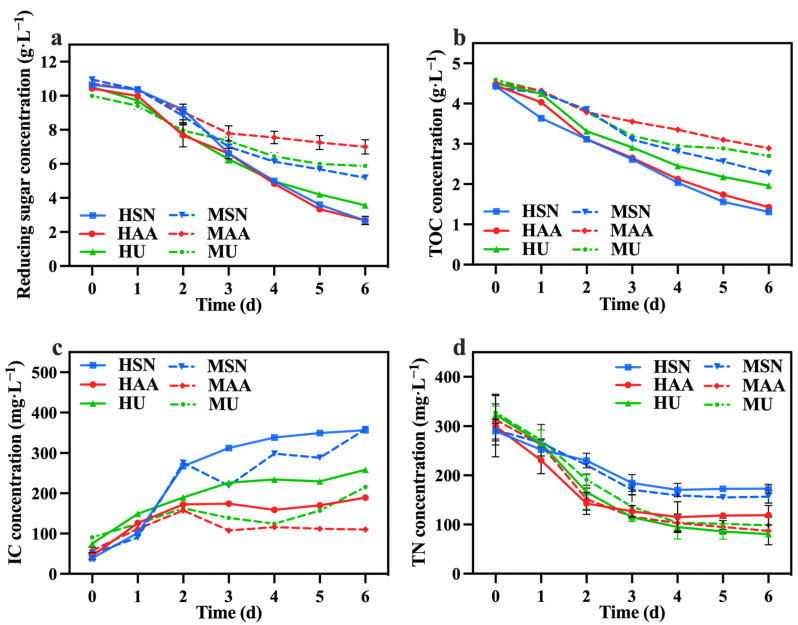
Reducing sugar concentration (**a**), IC concentration (**b**), TOC concentration (**c**), and TN concentration (**d**) in different groups with varying nitrogen sources and culture modes.

The variations in reducing sugar, TOC, IC, and TN concentrations among the different groups are shown in Figure 2a–d. The mixotrophic culture groups (MSN, MAA, MU) demonstrated lower rates of reducing sugar and TOC utilization in comparison to the heterotrophic culture groups (HSN, HAA, HU). This difference arose from the exclusive reliance of heterotrophic *C. vulgaris* on cellular respiration for metabolism and growth [23], while mixotrophic culture *C. vulgaris* could simultaneously perform photosynthesis and cellular respiration [24]. Consequently, *C. vulgaris* in the mixotrophic culture groups could achieve greater biomass production using a lesser amount of organic carbon through the synergistic effects of photosynthesis and cellular respiration [25] (also shown in Table 1). The groups supplemented with sodium nitrate (HSN, MSN) exhibited the highest rates of reducing sugar and TOC utilization. This observation indicates that sodium nitrate is more beneficial for *C. vulgaris* in terms of utilizing organic carbon compared with other nitrogen sources. The IC concentration in the media primarily originates from the carbon dioxide generated through the cellular respiration of *C. vulgaris*, thereby functioning as a measure of cellular respiration intensity. Analysis of IC concentration changes in Figure 2c indicates that the groups supplemented with sodium nitrate (HSN, MSN) displayed higher overall cellular respiration compared to the other groups, which is consistent with the observations on reducing sugar and TOC consumption.

**Figure 3:**
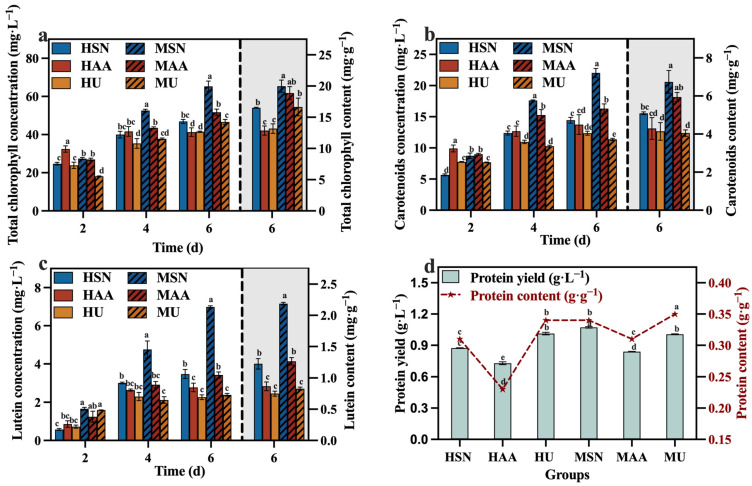
Total chlorophyll concentration (**a**), carotenoids concentration (**b**), lutein concentration (**c**) and protein yield (**d**) in different groups with varying nitrogen sources and culture modes (Different letters (a–e) represent significant differences (*p* < 0.05)).

The results in Figure 3a–c reveal that the groups supplemented with sodium nitrate (HSN, MSN) conferred greater advantages in the production of total chlorophyll, carotenoids, and lutein. In contrast, Del Campo, et al. [26] reported that the nitrogen sources (NaNO_3_, NH_4_Cl, and NH_4_NO_3_) did not influence the lutein level in *Muriellopsis* sp. It is speculated that the correlation between the nitrogen source and the lutein content may be species-specific. Meanwhile, the mixotrophic culture groups also demonstrated elevated levels of total chlorophyll, carotenoids, and lutein compared to the heterotrophic groups (Figure 3). The focus of this study is on lutein production. Figure 3c clearly shows that the concentration and content of lutein in the MSN group were significantly higher than the other groups, reaching 6.98 mg·L^−1^ and 2.19 mg·g^−1^, respectively. This outcome can be attributed to the influence of light conditions, which facilitate the synthesis of photosynthetic pigment. The protein content of the HU group with added urea was higher than that of the HSN group in the heterotrophic group, and this also applied to the MU and MSN groups. This finding aligns with the research by Batista, et al. [27], which demonstrated that microalgae can generate more protein when utilizing urea as a nitrogen source. However, combining the results of TN consumption (Figure 2d) and protein production (Figure 3d), the efficiencies of *C. vulgaris* in converting nitrogen sources into protein in the groups supplemented with sodium nitrate (HSN, MSN) are higher compared to the groups supplemented with urea (HU, MU). Moreover, the HSN group still had the highest protein production.

At the molecular level, central carbon metabolism pathways, including the tricarboxylic acid (TCA) cycle, pentose phosphate (PP) pathway, and Embden–Meyerhof pathway (EMP), significantly influence the provision of essential substrates and energy required for biomass accumulation and the biosynthesis of proteins and carotenoids. Variations and alterations in these pathways have a direct impact on product yields [28,29]. Consequently, it can be deduced that the mixotrophic cultivation of *C. vulgaris* using sodium nitrate as the nitrogen source enhanced respiratory action and promoted lutein content in *C. vulgaris* by elevating the carbon availability and metabolic flux within the central carbon metabolism, which supplied more substances and energy for the biosynthesis of products.

In conclusion, the MSN group demonstrated the most favorable performance. However, despite these optimized conditions, a substantial amount of reducing sugar, TOC, and TN remained in the media; consequently, further optimization is required to enhance the utilization by *C. vulgaris*.

### 3.2. Effect of Total-Organic-Carbon–Total-Nitrogen Ratio

**Figure 4:**
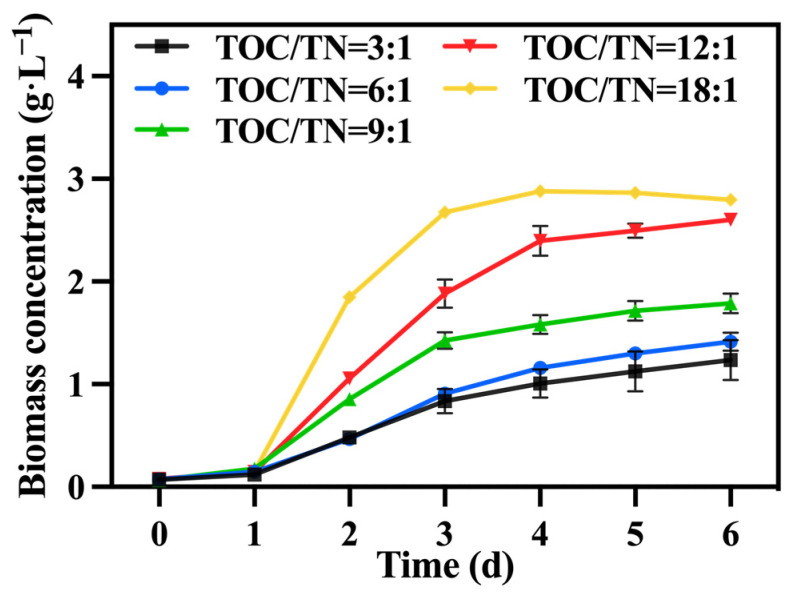
The biomass concentration in different groups with varying TOC/TN ratios.

Under the conditions of the preferred mixotrophic culture mode and sodium nitrate as the nitrogen source, the TOC/TN ratio was adjusted by varying the addition of sodium nitrate. Figure 4 shows the change in biomass concentration; it was observed that, under a fixed TOC concentration, a higher TOC/TN ratio led to a lower nitrogen level and higher biomass concentration. Generally, nitrogen is a vital component in the growth of *C. vulgaris*, and an increase in the nitrogen concentration typically promotes biomass and value-added compound accumulation. Hsieh and Wu [30] observed a significant enhancement in the growth of *Chlorella* sp. when the nitrogen concentration was increased from 0.025 to 0.2 g·L^−1^. However, certain studies have indicated that excessively high or low nitrogen concentrations are detrimental to biomass accumulation in *C. vulgaris*, with high nitrogen concentrations having toxic effects on it. According to Ye, et al. [31], when the nitrogen concentration reached excessive levels, the rate of intracellular nitrogen assimilation was notably lower than the rate of the nutrient supply; consequently, ammonium produced by the algal cells over-accumulated and could not be promptly utilized for amino acid synthesis, which subsequently affected the normal growth of microalgae. Therefore, in this study, it is speculated that nitrogen concentrations in the groups with TOC/TN ratios of 9:1, 6:1, and 3:1 exceeded the optimal range for *C. vulgaris* growth, resulting in adverse effects. Li, et al. [32] discovered that employing a high nitrate concentration for cultivating *Neochloris oleoabundans* resulted in the inhibition of cell growth. Table 2 demonstrates that the group with a TOC/TN ratio of 18:1 exhibited the highest biomass concentration and productivity, followed by the group with a TOC/TN ratio of 12:1. However, there was no significant difference between the two groups. This indicates that both the TOC/TN ratio of 12:1 and TOC/TN ratio of 18:1 groups are more suitable as culture mediums for *C. vulgaris*.

**Figure 5:**
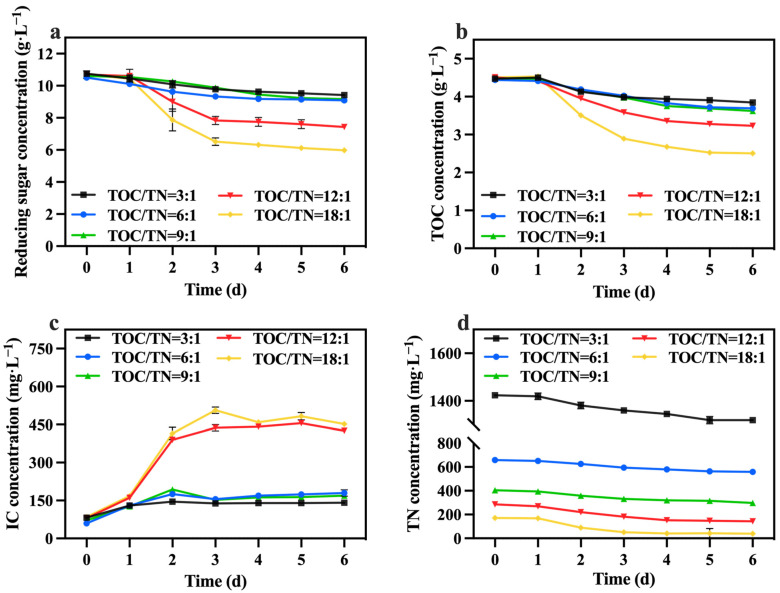
Reducing sugar concentration (**a**), IC concentration (**b**), TOC concentration (**c**) and TN concentration (**d**) in different groups with varying TOC/TN ratios.

The changes in reducing sugar and TOC concentrations are shown in Figure 5a,b. The utilization rate of reducing sugar and the TOC of each group was less than 50%, and the group with TOC/TN ratios of 18:1 exhibited the highest, followed by the group with a ratio of 12:1. The fluctuations in the IC concentration are presented in Figure 5c. The group with TOC/TN ratios of 18:1 and 12:1 showed significantly higher IC concentrations compared to the other groups. This observation indicates that the cellular respiration of *C. vulgaris* in these two groups was more robust than in the other groups, which is consistent with the consumption of reducing sugar and TOC. Table 2 reveals that the group with a ratio of 12:1 displayed the highest growth yield. Additionally, as shown in Figure 5d, the concentration of TN decreased by 88.80, 94.40, 100.40, 141.60, and 132.60 mg·L^−1^ in the groups with TOC/TN ratios of 3:1, 6:1, 9:1, 12:1, and 18:1, respectively. This finding demonstrates that higher nitrogen concentrations lead to reduced nitrogen consumption. The presence of excessive nitrogen concentrations poses challenges for *C. vulgaris* in efficiently utilizing these nitrogen sources. Meanwhile, excessive nitrogen concentrations can induce elevated salt content in the culture medium, heightened osmotic pressure, and cellular membrane impairment, resulting in a decrease in biomass [33,34].

**Figure 6:**
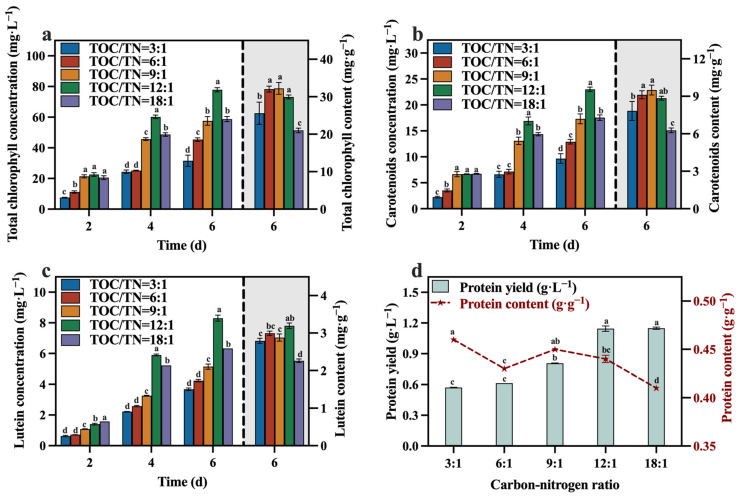
Total chlorophyll concentration (**a**), carotenoids concentration (**b**), lutein concentration (**c**) and protein yield (**d**) in different groups with varying TOC/TN ratios (Different letters (a–d) represent significant differences (*p* < 0.05)).

The concentrations and contents of total chlorophyll, carotenoids, lutein, and protein are depicted in Figure 6a–d. At the conclusion of the culture, the group with a TOC/TN ratio of 12:1 demonstrated significantly higher concentrations of total chlorophyll, carotenoids, and lutein compared to the other groups, while its protein yield was also at a higher level. In comparison to the optimized maximum concentrations of total chlorophyll (65.28 mg·L^−1^), carotenoids (22.04 mg·L^−1^), lutein (6.98 mg·L^−1^), and protein (1.07 g·L^−1^) in Section 3.1, selecting a media with a TOC/TN ratio of 12:1 can enhance the concentrations of total chlorophyll, carotenoids, lutein, and protein by 19.29%, 4.45%, 18.91%, and 6.54%, respectively. Meanwhile, it is worth noting that the group with a TOC/TN ratio of 12:1 exhibited the highest lutein content of 3.19 mg·g^−1^, which is 45.66% higher than the lutein content of 2.19 mg·g^−1^ optimized in Section 3.1. A related study indicated that nitrogen availability is a pivotal factor influencing the accumulation of lutein in microalgae throughout cultivation [35]. In addition, upon evaluating the protein yield of each group in conjunction with the corresponding TN consumption, it was observed that the conversion efficiency of the nitrogen source to protein was higher in the groups with TOC/TN ratios of 12:1 and 18:1 compared to the other groups.

Del Campo, et al. [26] observed that nitrogen limitation led to a reduction in lutein and biomass levels, while an appropriate increase in the sodium nitrate concentration facilitated lutein accumulation. The decrease in the TOC/TN ratio from 18:1 to 12:1 (indicating an increased nitrogen content) resulted in a significant increase in the lutein content in *C. vulgaris*. This phenomenon can be attributed to the essential role of nitrogen as a source for protein synthesis, with lutein existing as a nitrogenous macromolecule in microalgae. Under nitrogen-depleted conditions, the nitrogenous macromolecules accumulated inside the microalgal cells may undergo transformation into lipids or carbohydrates [36]. Hence, this leads to an increase in the lutein content upon proper nitrogen supplementation. However, the lutein content did not exhibit a sustained increase as the TOC/TN ratio continued to decrease (Figure 6c). Similar results were reported for *Desmodesmus* sp. [37], *Scenedesmus obliquus* FSP-3 [38], and *C. sorokiniana* 211-32 [39]. This may be attributed to the excessive nitrogen resulting in a low biomass of *C. vulgaris*, leading to a decline in lutein production. Based on the literature [40,41], it appears that lutein is considered a growth-coupled metabolite, indicating that the production of lutein is closely linked to cell growth. Therefore, optimizing the growth conditions of *C. vulgaris* is vital to promoting its lutein accumulation [39].

The rate of nitrogen absorption is closely intertwined with the rate of carbon metabolism [42]. Although the TOC/TN ratio was optimized, both TOC and TN levels remained elevated in each group. This observation suggests the existence of additional factors that limit the rate of carbon metabolism. Therefore, further optimization of culture conditions is imperative to augment the rate of TOC utilization and biomass accumulation by *C. vulgaris*.

### 3.3. Effect of Total-Nitrogen–Total-Phosphorus Ratio

**Figure 7:**
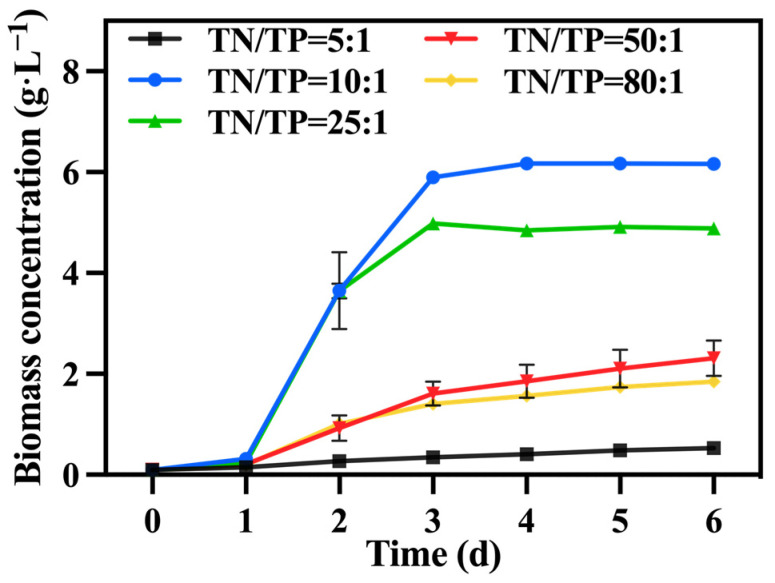
The biomass concentration in different groups with varying TN/TP ratios.

In the preferred mixed culture mode described in Section 3.1 and Section 3.2, sodium nitrate was utilized as the nitrogen source while maintaining a TOC/TN ratio of 12:1. The TN/TP ratio was regulated by adjusting the quantity of added phosphorus source (K_2_HPO_4_), while the levels of TOC and TN were kept constant within each group. Figure 7 illustrates the variations in biomass accumulation of *C. vulgaris*. The biomass accumulation of *C. vulgaris* gradually increased as the TN/TP ratio decreased from 80:1 to 10:1. However, a further decrease to a ratio of TN/TP of 5:1 resulted in a steep decline in biomass accumulation. Phosphorus directly influences the absorption efficiency of carbon and nitrogen in microalgae, consequently impacting its biomass production and nutrient accumulation [43]. Therefore, an increase in the phosphorus addition led to a higher final biomass accumulation in *C. vulgaris*. However, the excessive addition of phosphorus sources became problematic when the TN/TP ratio decreased from 10:1 to 5:1, as it could have had toxic effects [44]. An appropriate phosphorus concentration (TN/TP ratio) was conducive to enhancing the growth and nutrient assimilation of the algal cells [45]. The data presented in Table 3 highlight a significant increase in the final biomass accumulation and productivity of the group with a TN/TP ratio of 10:1 compared to the other groups, reaching 6.61 g·L^−1^ and 1.01 g·L^−1^·d^−1^, respectively. Compared to the optimized maximum biomass accumulation (2.6 g·L^−1^) and productivity (0.42 g·L^−1^·d^−1^) achieved in Section 3.2, selecting a medium with a TN/TP ratio of 10:1 can enhance the biomass accumulation and productivity of *C. vulgaris* by 237.00% and 240.00%, respectively.

**Figure 8:**
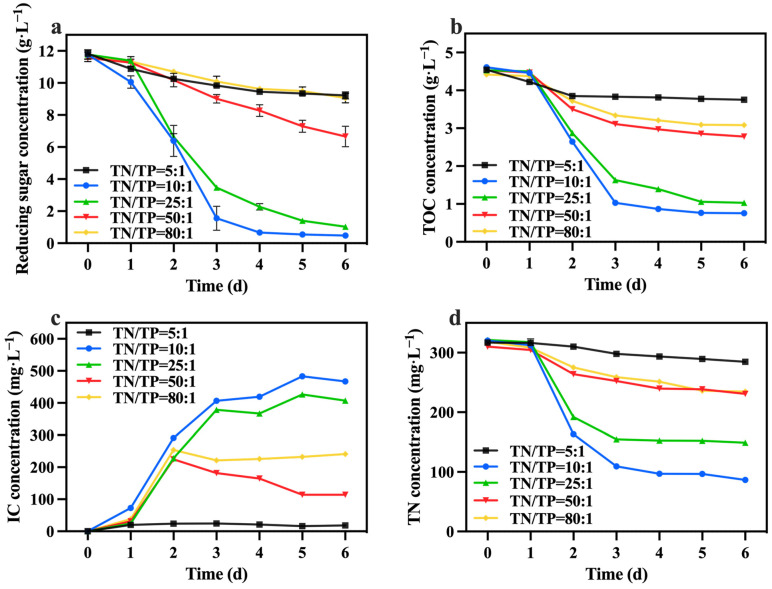
Reducing sugar concentration (**a**), IC concentration (**b**), TOC concentration (**c**) and TN concentration (**d**) in different groups with varying TN/TP ratios.

Figure 8a,b shows the change in reducing sugar and TOC concentrations. The group with a TN/TP ratio of 10:1 exhibited the highest consumption of reducing sugar and TOC, with utilization rates of 96.01% and 83.57%, respectively, greater than those of Section 3.2. According to Table 3, the group with a TN/TP ratio of 10:1 achieved the highest growth yield, reaching 1.60 g biomass/g TOC. The carbon metabolism in microalgae relies on phosphorus-dependent transformation pathways, including the generation and consumption of ATP. It is speculated that increasing the phosphorus concentration can significantly enhance the utilization of reducing sugar and TOC by *C. vulgaris*, thereby promoting biomass accumulation. Figure 8c presents the IC concentration, showing that the group with a TN/TP ratio of 10:1 exhibited a higher IC concentration compared to the other groups. This trend was consistent with the change in reducing sugar and TOC consumption rates. This indicates that the cellular respiration of *C. vulgaris* was more robust in the group with a TN/TP ratio of 10:1 compared to the other groups. Furthermore, Figure 8d reveals that the group with a TN/TP ratio of 10:1 exhibited the highest TN consumption, reaching 262.6 mg·L^−1^. Phosphorus concentration has a significant effect on nitrogen utilization, thereby impacting both microalgal cell growth and metabolism [45]. Due to the correlation between nitrogen metabolism and carbon metabolism in *C. vulgaris*, an elevation in the carbon metabolism rate of *C. vulgaris* corresponded to an augmentation in the rate of nitrogen metabolism within the same microorganism.

**Figure 9:**
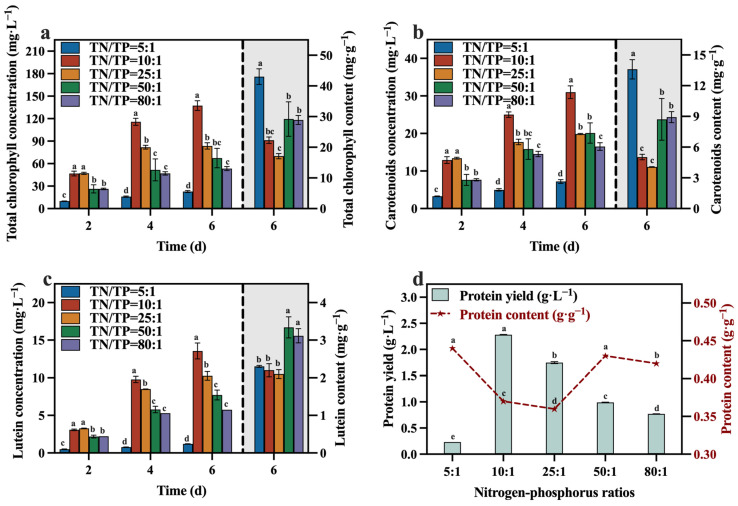
Total chlorophyll concentration (**a**), carotenoids concentration (**b**), lutein concentration (**c**) and protein yield (**d**) in different groups with varying TN/TP ratios (Different letters (a–e) represent significant differences (*p* < 0.05)).

The concentrations and contents of total chlorophyll, carotenoid, lutein, and protein during the culture process are depicted in Figure 9a–d. At the conclusion of the culture, the group with a TN/TP ratio of 10:1 contained lower levels of total chlorophyll, carotenoid, lutein, and protein compared to the other groups (except the group with a TN/TP ratio of 25:1). This may be attributed to a significant increase in biomass within the TN/TP ratio of the 10:1 group (Figure 7), resulting in the formation of light shielding between algal cells, leading to a low level of photosynthetic efficiency and lutein synthesis. Despite these observations, the group with a TN/TP ratio of 10:1 exhibited remarkable advantages in terms of total chlorophyll, carotenoid, lutein, and protein yields, surpassing the other groups, reaching 137.40 mg·L^−1^, 30.99 mg·L^−1^, 13.56 mg·L^−1^, and 2.28 g·L^−1^, respectively. These values represent a substantial increase in the concentrations, as optimized in Section 3.2, with concentration enhancements of 76.45%, 34.62%, 62.98%, and 100.00%, respectively. This may be due to the higher biomass production in the TN/TP ratio of the 10:1 group, resulting in significantly higher concentrations of value-added compounds, including lutein. Indeed, after optimizing the TN/TP ratio, significant improvements were observed in the accumulation of biomass and value-added compounds, as well as the utilization rates of reducing sugar, TOC, and TN in the medium.

Researchers have employed a combination of cyclic autotrophic and heterotrophic cultivation to enhance the accumulation of lutein in microalgae [46]. The abrupt transition of cultivation methods induced by alternating light and dark cycles has the potential to impose stress on algal cells, thereby stimulating the synthesis of pigment-like compounds. Consequently, to enhance lutein production, investigations were conducted to examine the influence of light duration on *C. vulgaris*.

### 3.4. Effect of Lighting Duration

**Figure 10:**
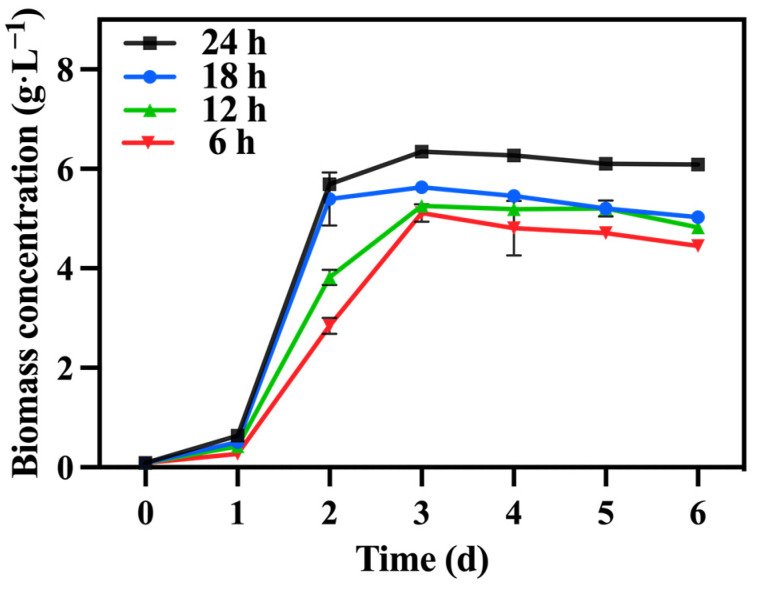
The biomass concentration in different groups with varying lighting durations.

Under the optimized conditions described above, involving mixotrophic cultivation with sodium nitrate as the nitrogen source and a TOC/TN ratio of 12:1 and a TN/TP ratio of 10:1, the duration of lighting varied while maintaining a light intensity of 10,000 lux across all groups. Table 4 reveals that the 24 h group exhibited a significantly higher biomass concentration and productivity compared to the other groups, reaching 6.08 g·L^−1^ and 1.00 g·L^−1^·day^−1^, respectively. Notably, a shorter lighting duration led to reduced biomass accumulation (Figure 10).

**Figure 11:**
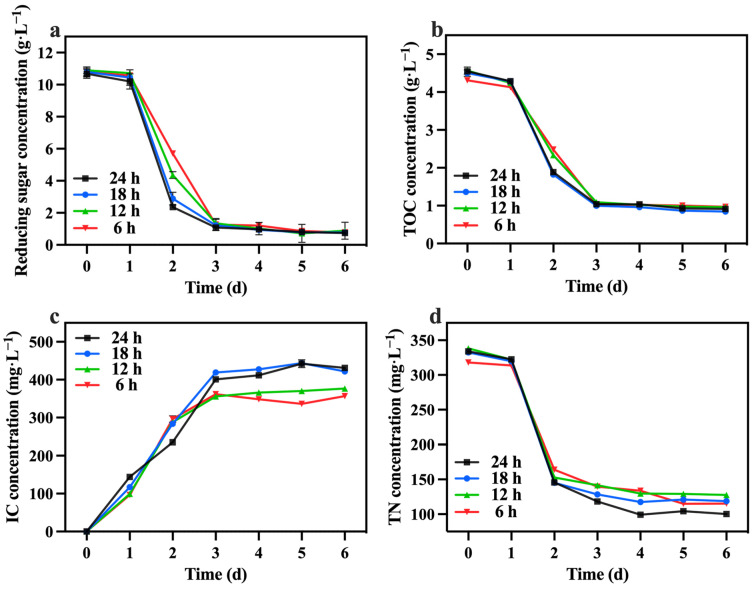
Reducing sugar concentration (**a**), IC concentration (**b**), TOC concentration (**c**) and TN concentration (**d**) in different groups with varying lighting durations.

The changes in reducing sugar and TOC concentrations are presented in Figure 11a,b. No obvious differences were observed in the consumption of reducing sugars and TOC among the groups. However, it is worth noting that the consumption of reducing sugars exceeded 90% in all groups. Consequently, groups with lighting durations shorter than 24 h were unable to generate sufficient energy in the dark, resulting in the mortality of certain algal cells. Table 4 highlights that the 24 h lighting duration group exhibited the highest growth yield (1.67 g biomass/g TOC). The variations in the IC concentration are illustrated in Figure 11c. The IC concentration in the 24 h and 18 h groups were higher compared to the 12 h and 6 h groups. This observation suggests that the alternating light and dark periods strategy influenced the respiratory intensity of *C. vulgaris*, with a decrease in respiratory intensity occurring when the dark period exceeded 12 h. Additionally, as depicted in Figure 11d, the 24 h group demonstrated the highest TN consumption, reaching 233.80 mg·L^−1^. This could be attributed to insufficient reducing sugars, which hindered the nitrogen source’s metabolism during the dark period.

**Figure 12:**
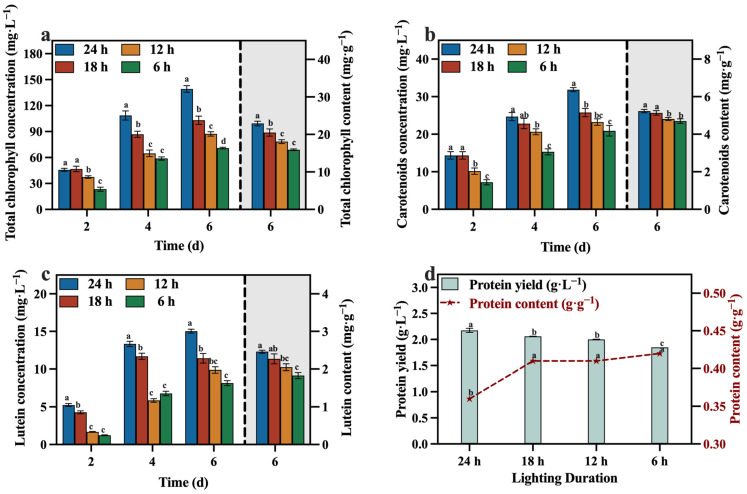
Total chlorophyll concentration (**a**), carotenoids concentration (**b**), lutein concentration (**c**) and protein yield (**d**) in different groups with varying lighting durations (Different letters (a–d) represent significant differences (*p* < 0.05)).

The concentrations and contents of total chlorophyll, carotenoids, lutein, and protein are presented in Figure 12a–d. The 24 h group exhibited higher concentrations and contents of these value-added compounds compared to the other groups, especially the concentration of lutein, reaching 15.02 mg·L^−1^. And reducing the duration of light exposure had adverse effects on both the growth of *C. vulgaris* and the synthesis of value-added compounds. This was attributed to the inability of microalgae to carry out photosynthesis in the absence of light and their incapacity to sustain heterotrophic growth during the later phases of cultivation, owing to the depletion of reducing sugars. Consequently, not only does this impede the overall growth rate, but it also significantly hampers the production of valuable compounds, specifically lutein.

### 3.5. Principal Component Analysis

**Figure 13:**
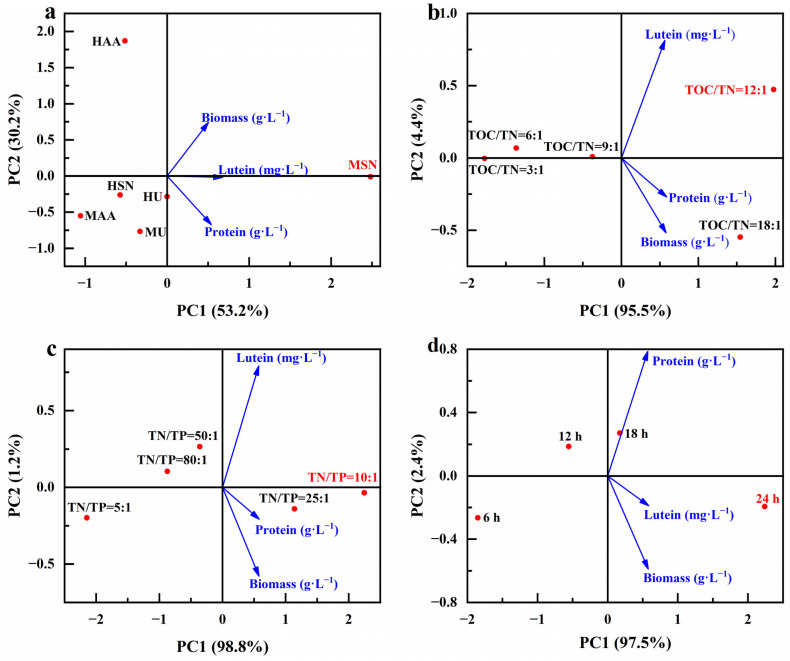
Principal component analysis diagram of different optimization conditions (culture mode and nitrogen source (**a**), TOC/TN (**b**), TN/TP (**c**) and lighting duration (**d**)), microalgae biomass growth and the production of value-added compounds.

Here, we examined the correlation between the parameters related to microalgae biomass growth and parameters related to the production of value-added compounds of different optimized conditions (Figure 13). It is clearly shown that sodium nitrate as a nitrogen source under mixed cultivation conditions (MSN) was associated with higher biomass and lutein and protein concentrations (Figure 13a). Adjustments to a TOC/TN ratio of 12:1 or a TN/TP ratio of 10:1 are linked to heightened biomass and lutein and protein concentrations (Figure 13b,c). It is noteworthy that diverse optimization conditions share a common aspect: the lutein concentration is closely intertwined with biomass production. This observation aligns with the speculated results outcomes in Section 3.2; lutein is a growth-coupled metabolite, and the production of lutein is closely related to the growth of microalgae.

## 4. Conclusions

In this study, the final optimization conditions of culturing *Chlorella vulgaris* to accumulate biomass and lutein were achieved by using a mixed culture with sodium nitrate as the nitrogen source, adjusting for the total organic carbon: total nitrogen = 12:1; adjusting for the total nitrogen: total phosphorus = 10:1; controlling the light time for 24 h. These conditions dramatically increased the efficiency of the carbon and nitrogen metabolism of *Chlorella vulgaris*, which led to an increase in the utilization rate of nutrients in the culture medium. Thus, the biomass productivity of *Chlorella vulgaris* and the production of various nutrients, including lutein, were increased. The findings of the study provide technical guidance for the industrial applications of microalgae in lutein production. In the future, based on the optimal conditions of this study, we will search for alternative carbon sources of glucose to further compress the culture cost and further process the obtained *Chlorella vulgaris* into microalgae food.

## Figures and Tables

**Table 1:** Growth kinetic parameters of *Chlorella vulgaris* in different groups with varying nitrogen sources and culture modes.

Groups	μ Max (day^−1^)	Generation Time (days)	Biomass Productivity (g·L^−1^·day^−1^)	Biomass Concentration (g·L^−1^)	Growth Yield (g Biomass/g TOC)
HSN	1.81 ± 0.04 ^b^	0.38 ± 0.01 ^cd^	0.45 ± 0.01 ^a^	2.84 ± 0.07 ^a^	0.94 ± 0.02 ^c^
HAA	1.60 ± 0.07 ^bc^	0.43 ± 0.02 ^abc^	0.51 ± 0.01 ^a^	3.21 ± 0.08 ^a^	1.02 ± 0.03 ^bc^
HU	1.63 ± 0.08 ^bc^	0.43 ± 0.02 ^bc^	0.49 ± 0.08 ^a^	3.02 ± 0.46 ^a^	1.19 ± 0.18 ^b^
MSN	2.10 ± 0.37 ^a^	0.34 ± 0.06 ^d^	0.51 ± 0.04 ^a^	3.19 ± 0.25 ^a^	1.50 ± 0.12 ^a^
MAA	1.52 ± 0.05 ^bc^	0.46 ± 0.02 ^ab^	0.43 ± 0.04 ^a^	2.71 ± 0.25 ^a^	1.67 ± 0.15 ^a^
MU	1.46 ± 0.05 ^c^	0.48 ± 0.02 ^a^	0.46 ± 0.04 ^a^	2.88 ± 0.20 ^a^	1.53 ± 0.10 ^a^

Values in the same column with different superscripts (a–d) are significantly different (*p* < 0.05).

**Table 2:** Growth kinetic parameters of *C. vulgaris* in different groups with varying TOC/TN ratios.

Groups	μ Max (day^−1^)	Generation Time (days)	Biomass Productivity (g·L^−1^·day^−1^)	Biomass Concentration (g·L^−1^)	Growth Yield (g Biomass/g TOC)
TOC/TN = 3:1	1.38 ± 0.03 ^d^	0.50 ± 0.01 ^b^	0.19 ± 0.04 ^c^	1.23 ± 0.24 ^c^	1.96 ± 0.38 ^a^
TOC/TN = 6:1	1.14 ± 0.08 ^e^	0.61 ± 0.04 ^a^	0.22 ± 0.02 ^c^	1.41 ± 0.11 ^c^	1.88 ± 0.15 ^a^
TOC/TN = 9:1	1.61 ± 0.04 ^c^	0.43 ± 0.01 ^c^	0.29 ± 0.02 ^b^	1.79 ± 0.12 ^b^	2.09 ± 0.14 ^a^
TOC/TN = 12:1	1.96 ± 0.04 ^b^	0.35 ± 0.01 ^d^	0.42 ± 0.01 ^a^	2.60 ± 0.06 ^a^	2.03 ± 0.05 ^a^
TOC/TN = 18:1	2.52 ± 0.03 ^a^	0.27 ± 0.01 ^e^	0.45 ± 0.01 ^a^	2.80 ± 0.06 ^a^	1.40 ± 0.03 ^b^

Values in same column with different superscripts (a–e) are significantly different (*p* < 0.05).

**Table 3:** Growth kinetic parameters of *C. vulgaris* in different groups with varying TN/TP ratios.

Groups	μ Max (day^−1^)	Generation Time (days)	Biomass Productivity (g·L^−1^·day^−1^)	Biomass Concentration (g·L^−1^)	Growth Yield (g Biomass/g TOC)
TN/TP = 5:1	0.58 ± 0.01 ^d^	1.19 ± 0.02 ^a^	0.07 ± 0.00 ^e^	0.53 ± 0.01 ^e^	0.67 ± 0.01 ^c^
TN/TP = 10:1	2.44 ± 0.01 ^b^	0.28 ± 0.00 ^c^	1.01 ± 0.01 ^a^	6.16 ± 0.02 ^a^	1.60 ± 0.01 ^a^
TN/TP = 25:1	2.73 ± 0.02 ^a^	0.25 ± 0.01 ^c^	0.80 ± 0.02 ^b^	4.89 ± 0.13 ^b^	1.40 ± 0.04 ^b^
TN/TP = 50:1	1.45 ± 0.33 ^c^	0.50 ± 0.13 ^b^	0.37 ± 0.07 ^c^	2.31 ± 0.43 ^c^	1.32 ± 0.24 ^b^
TN/TP = 80:1	1.64 ± 0.13 ^c^	0.43 ± 0.03 ^b^	0.29 ± 0.01 ^d^	1.85 ± 0.06 ^d^	1.28 ± 0.04 ^b^

Values in the same column with different superscripts (a–e) are significantly different (*p* < 0.05).

**Table 4:** Growth kinetic parameters of *C. vulgaris* in different groups with varying lighting durations.

Groups	μ Max (day^−1^)	Generation Time (days)	Biomass Productivity (g·L^−1^·day^−1^)	Biomass Concentration (g·L^−1^)	Growth Yield (g Biomass/g TOC)
24 h	2.19 ± 0.04 ^b^	0.32 ± 0.01 ^a^	1.00 ± 0.03 ^a^	6.09 ± 0.14 ^a^	1.67 ± 0.04 ^a^
18 h	2.37 ± 0.11 ^a^	0.30 ± 0.02 ^a^	0.82 ± 0.02 ^b^	5.03 ± 0.14 ^b^	1.37 ± 0.04 ^b^
12 h	2.20 ± 0.03 ^b^	0.31 ± 0.01 ^a^	0.79 ± 0.01 ^c^	4.83 ± 0.07 ^c^	1.34 ± 0.02 ^b^
6 h	2.36 ± 0.10 ^a^	0.30 ± 0.02 ^a^	0.73 ± 0.01 ^d^	4.45 ± 0.06 ^d^	1.33 ± 0.01 ^b^

Values in the same column with different superscripts (a–d) are significantly different (*p* < 0.05).

## Data Availability

The data presented in this study are available on request from the corresponding author due to privacy.

## Supplementary Materials

The following supporting information can be downloaded at https://www.mdpi.com/article/10.3390/foods13162514/s1, Figure S1: Linear relationship between optical density of *Chlorella vulgaris* and biomass dry weight; Table S1: Medium composition; Table S2: The compositions of adjusted medium in different nitrogen sources groups; Table S3: The compositions of adjusted medium in different TOC/TN groups; Table S4: The compositions of adjusted medium in different TN/TP groups.
