# Supplementary material for: Optimizing Chlorella vulgaris Cultivation to Enhance Biomass and Lutein Production
Source: Foods. 2024 Aug 12;13(16):2514. doi: 10.3390/foods13162514 (PMC11353733; doi:10.3390/foods13162514)
Supplement: Supplementary file 1 [file foods-13-02514-s001.zip › foods-3124564-supplementary.pdf]

Supplementary material

Optimizing *Chlorella vulgaris* Cultivation to Enhance Biomass and Lutein Production

Kangping Wu [1,2,†], Jiangling Lai [1,†], Qi Zhang [1,*], Yunpu Wang [1], Xian Cui [1], Yuhuan Liu [3,*], Xiaodan Wu [1], Zhigang Yu [4], Roger Ruan [5].

[1] State Key Laboratory of Food Science and Resources, Engineering Research Center for Biomass Conversion, Ministry of Education, Nanchang University, Nanchang 330047, China.

[2] School of Resources and Civil Engineering, Gannan University of Science and Technology, Ganzhou 341000, China.

[3] College of Food Science and Technology, Nanchang University, Nanchang 330047, China

[4] Australian Centre for Water and Environmental Biotechnology (formerly AWMC), The University of Queens-land, Brisbane QLD4072 Australia.

[5] Center for Biorefining and Department of Bioproducts and Biosystems Engineering, University of Minnesota, St Paul, MN 55108, USA

[†] These authors contributed equally to this work.

* Corresponding authors: zhangqi093115@ncu.edu.cn (Q. Zhang); liuyuhuan@ncu.edu.cn (Y. Liu)

Supporting Information content:

Number of pages: 6

Number of tables: 4

Number of figures:1

Supplementary material

Table S1 Medium composition

Ingredient	BG-11 supplemented with glucose
**Nitrogen source and mineral**	
$NaNO_3$ ($g \cdot L^{-1}$)	1.500
K_2HPO_4 ($g \cdot L^{-1}$)	0.040
$MgSO_4 \cdot 7H_2O$ ($g \cdot L^{-1}$)	0.075
$CaCl_2 \cdot 2H_2O$ ($g \cdot L^{-1}$)	0.036
Citric acid ($g \cdot L^{-1}$)	0.006
Ferric ammonium citrate ($g \cdot L^{-1}$)	0.006
EDTA·2Na ($g \cdot L^{-1}$)	0.001
Na_2CO_3 ($g \cdot L^{-1}$)	0.020
A5 ($mg \cdot L^{-1}$) [a]	1.000
**Carbon source**	
Glucose ($g \cdot L^{-1}$)	10.000

[a] The concentrations of H_3BO_3, $MnCl_2 \cdot 4H_2O$, $ZnSO_4 \cdot 7H_2O$, $NaMoO_4 \cdot 2H_2O$, $CuSO_4 \cdot 5H_2O$ and $Co(NO_3)_2 \cdot 6H_2O$ in A5 solution were 2.86, 1.81, 0.22, 0.39, 0.08 and 0.05 $g \cdot L^{-1}$, respectively.

Table S2 The compositions of adjusted medium in different nitrogen sources groups

Ingredient	HSN	HAA	HU	MSN	MAA	MU
**Nitrogen source**						
$NaNO_3$ ($g \cdot L^{-1}$)	1.500			1.500		
CH_3COONH_4 ($g \cdot L^{-1}$)		1.361			1.361	
Urea ($g \cdot L^{-1}$)			0.530			0.530
CH_3COONa ($g \cdot L^{-1}$)	1.447		1.447	1.447		1.447
**Carbon source**						
Glucose ($g \cdot L^{-1}$)	10.000	10.000	10.000	10.000	10.000	10.000
**Phosphorus source**						
K_2HPO_4 ($g \cdot L^{-1}$)	0.040	0.040	0.040	0.040	0.040	0.040
**Minerals**						
$MgSO_4 \cdot 7H_2O$ ($g \cdot L^{-1}$)	0.075	0.075	0.075	0.075	0.075	0.075
$CaCl_2 \cdot 2H_2O$ ($g \cdot L^{-1}$)	0.036	0.036	0.036	0.036	0.036	0.036
Citric acid ($g \cdot L^{-1}$)	0.006	0.006	0.006	0.006	0.006	0.006
Ferric ammonium citrate ($g \cdot L^{-1}$)	0.006	0.006	0.006	0.006	0.006	0.006
EDTA·2Na ($g \cdot L^{-1}$)	0.001	0.001	0.001	0.001	0.001	0.001
Na_2CO_3 ($g \cdot L^{-1}$)	0.020	0.020	0.020	0.020	0.020	0.020
A5 ($mg \cdot L^{-1}$) [a]	1.000	1.000	1.000	1.000	1.000	1.000

[a] The concentrations of H_3BO_3, $MnCl_2 \cdot 4H_2O$, $ZnSO_4 \cdot 7H_2O$, $NaMoO_4 \cdot 2H_2O$, $CuSO_4 \cdot 5H_2O$ and $Co(NO_3)_2 \cdot 6H_2O$ in A5 solution were 2.86, 1.81, 0.22, 0.39, 0.08 and 0.05 $g \cdot L^{-1}$, respectively.

Table S3 The compositions of adjusted medium in different TOC/TN groups

**Ingredient**	**3:1**	**6:1**	**9:1**	**12:1**	**18:1**
**Nitrogen source**					
$NaNO_3$ ($g{\cdot}L^{-1}$)	8.936	4.468	2.979	2.234	1.500
CH_3COONa ($g{\cdot}L^{-1}$)	1.447	1.447	1.447	1.447	1.447
**Carbon source**					
Glucose ($g{\cdot}L^{-1}$)	10.000	10.000	10.000	10.000	10.000
**Phosphorus source**					
K_2HPO_4 ($g{\cdot}L^{-1}$)	0.040	0.040	0.040	0.040	0.040
**Minerals**					
$MgSO_4{\cdot}7H_2O$ ($g{\cdot}L^{-1}$)	0.075	0.075	0.075	0.075	0.075
$CaCl_2{\cdot}2H_2O$ ($g{\cdot}L^{-1}$)	0.036	0.036	0.036	0.036	0.036
Citric acid ($g{\cdot}L^{-1}$)	0.006	0.006	0.006	0.006	0.006
Ferric ammonium citrate ($g{\cdot}L^{-1}$)	0.006	0.006	0.006	0.006	0.006
EDTA·2Na ($g{\cdot}L^{-1}$)	0.001	0.001	0.001	0.001	0.001
Na_2CO_3 ($g{\cdot}L^{-1}$)	0.020	0.020	0.020	0.020	0.020
A5 ($mg{\cdot}L^{-1}$) [a]	1.000	1.000	1.000	1.000	1.000

[a] The concentrations of H_3BO_3, $MnCl_2{\cdot}4H_2O$, $ZnSO_4{\cdot}7H_2O$, $NaMoO_4{\cdot}2H_2O$, $CuSO_4{\cdot}5H_2O$ and $Co(NO_3)_2{\cdot}6H_2O$ in A5 solution were 2.86, 1.81, 0.22, 0.39, 0.08 and 0.05 $g{\cdot}L^{-1}$, respectively.

Table S4 The compositions of adjusted medium in different TN/TP groups

**Ingredient**	**5:1**	**10:1**	**25:1**	**50:1**	**80:1**
**Nitrogen source**					
$NaNO_3$ ($g \cdot L^{-1}$)	2.234	2.234	2.234	2.234	2.234
CH_3COONa ($g \cdot L^{-1}$)	1.447	1.447	1.447	1.447	1.447
**Carbon source**					
Glucose ($g \cdot L^{-1}$)	10.000	10.000	10.000	10.000	10.000
**Phosphorus source**					
K_2HPO_4 ($g \cdot L^{-1}$)	0.414	0.207	0.083	0.041	0.026
**Minerals**					
$MgSO_4 \cdot 7H_2O$ ($g \cdot L^{-1}$)	0.075	0.075	0.075	0.075	0.075
$CaCl_2 \cdot 2H_2O$ ($g \cdot L^{-1}$)	0.036	0.036	0.036	0.036	0.036
Citric acid ($g \cdot L^{-1}$)	0.006	0.006	0.006	0.006	0.006
Ferric ammonium citrate ($g \cdot L^{-1}$)	0.006	0.006	0.006	0.006	0.006
EDTA·2Na ($g \cdot L^{-1}$)	0.001	0.001	0.001	0.001	0.001
Na_2CO_3 ($g \cdot L^{-1}$)	0.020	0.020	0.020	0.020	0.020
A5 ($mg \cdot L^{-1}$) [a]	1.000	1.000	1.000	1.000	1.000

[a] The concentrations of H_3BO_3, $MnCl_2 \cdot 4H_2O$, $ZnSO_4 \cdot 7H_2O$, $NaMoO_4 \cdot 2H_2O$, $CuSO_4 \cdot 5H_2O$ and $Co(NO_3)_2 \cdot 6H_2O$ in A5 solution were 2.86, 1.81, 0.22, 0.39, 0.08 and 0.05 $g \cdot L^{-1}$, respectively.

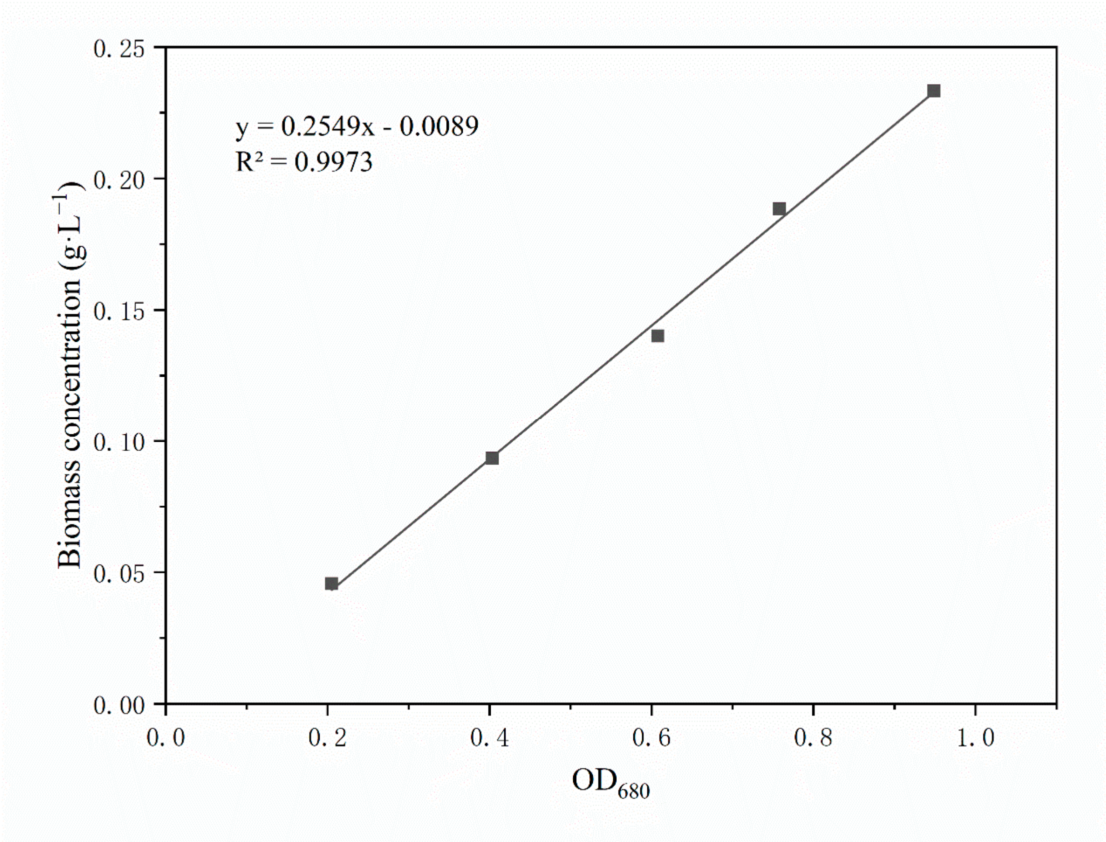

Figure S1 Linear relationship between optical density of *Chlorella vulgaris* and biomass dry weight
